# Autophagy Inhibition Contributes to Apoptosis of PLK4 Downregulation-induced Dormant Cells in Colorectal Cancer

**DOI:** 10.7150/ijbs.79949

**Published:** 2023-05-29

**Authors:** Xiangdong Tian, Yuchao He, Lisha Qi, Dongming Liu, Dejun Zhou, Yun Liu, Wenchen Gong, Zhiqiang Han, Yuren Xia, Hua Li, Jiefu Wang, Kangwei Zhu, Lu Chen, Hua Guo, Qiang Zhao

**Affiliations:** National Clinical Research Center for Cancer, Key Laboratory of Cancer Prevention and Therapy, Tianjin's Clinical Research Center for Cancer, Tianjin Medical University Cancer Institute and Hospital, Tianjin 300060, China.

**Keywords:** autophagy, dormancy, PLK4, colorectal cancer

## Abstract

Dormant cancer cells account for cancer recurrence, distant metastasis and drug resistance which lead to poor prognosis in colorectal cancer (CRC). However, little is known about the molecular mechanisms regulating tumor cell dormancy and how to eliminate dormant cancer cells. Recent studies indicate autophagy affects dormant tumor cell survival. Here, we found that polo-like kinases 4 (PLK4), a central regulator of the cell cycle and proliferation, plays a crucial role in regulating CRC cells dormancy both *in vitro* and in *vivo*. Downregulation of PLK4 induced dormancy and inhibited migration and invasion in different CRC cell lines. Clinically, PLK4 expression was correlated with the dormancy markers (Ki67, p-ERK, p-p38) and late recurrence in CRC tissues. Mechanistically, downregulation of PLK4 induced autophagy contributed to restoring phenotypically aggressive tumor cells to a dormant state through the MAPK signaling pathway, and inhibition of autophagy would trigger apoptosis of dormant cells. Our findings reveal that downregulation of PLK4-induced autophagy contributes to tumor dormancy and autophagy inhibition leads to apoptosis of CRC dormant cells. Our study is the first to report that downregulation PLK4 induced autophagy is an early event in CRC dormancy and highlights autophagy inhibitor as a potential therapeutic target for dormant cell elimination.

## Introduction

CRC is one of the most commonly diagnosed cancers among men and women worldwide^1^. The US 2021 cancer statistics report shows that both the morbidity and mortality of CRC rank in the top five among those of all cancer types^2^. Local recurrence and distant metastasis account for the great majority of cancer-related deaths for CRC patients, especially those with advanced CRC^3^. Nearly one-third of CRC cases at diagnosis already have disseminated disease lodged in target organs, such as the liver and lymph nodes, and 25-40% of patients with CRC will experience local or distant metastatic relapse, sometimes years or even decades after apparent cure^4^, ^5^.

Recurrence and metastasis usually originate from residual and disseminated tumor cells. Previous studies have shown that a subpopulation of disseminated tumor cells can escape radiotherapy, chemotherapy and immune monitoring or developing a "dormant" phenotype^6^, ^7^. These dormant tumor cells can be long-term latent in target organs with a state of balanced proliferation or no proliferation at all which are clinically undetectable, and patients usually have no clinical manifestations or symptoms^8^-^10^. When dormant tumor cells are reactivated and reenter the cell cycle, cancer recurrence occurs^7^. Thus, it is imperative to further elucidate the molecular mechanism of CRC dormancy, develop novel therapeutic strategies to target dormant cells and reduce the mortality of this malignancy.

Abundant evidence has demonstrated that autophagy, a core molecular pathway for the preservation of cellular and organismal homeostasis, plays an important role in the adaptation, survival and reactivation of dormant cells^11^. Nowadays, autophagy activation was reported as a novel characteristic of dormant cells in different cancer types and autophagy was regarded as an important mechanism contributing to cancer dormancy^12^. Nevertheless, the role of autophagy in the regulation of dormancy has been poorly understood in CRC. Given that autophagy activation is observed in various models of individual dormant cancer cells, a more detailed knowledge of the effect of autophagy inhibitor on dormancy is necessary to develop novel agents against dormant CRC cells.

To date, proteins that regulate cell cycle reprogramming have been fairly well studied. Among these proteins, PLK4, a member of the Polo-like kinase family, was identified as an important regulator of cell cycle-related events^13^, ^14^. Due to the critical role of PLK4 in controlling cell cycle progression, its importance in oncogenesis can be hypothesized^15^, ^16^. However, regulating cell proliferation and controlling the cell cycle may not be the only functions of PLK4 relevant to tumorigenicity. The potential association between PLK4 and autophagic activity needs to be further explored.

In the current study, we first verified knockdown PLK4 impaired proliferation, which was accompanied by G0/G1 cell cycle arrest and low Ki67 expression, characteristics of dormancy. We further authenticated that it was downregulation of PLK4-induced autophagy that contributed to restoring phenotypically aggressive tumor cells to the dormant state through the MAPK signaling pathway and inhibition of autophagy would trigger apoptosis of dormant cell and facilitate the dormant cells elimination.

## Materials and Methods

### Patients and tissue specimens

Tissue samples were tested and verified as CRC or adjacent non-tumor tissue by two independent pathologists, and the CRC samples were staged based on the 8th edition of the American Joint Committee on Cancer (AJCC) Cancer Staging Manual. This study was approved by the Ethics Committee of Tianjin Medical University Cancer Institute and Hospital and was consistent with the ethical guidelines of the Helsinki Declaration.

### Cell transfection

To acquire lentiviral particles, expression plasmids (sh-control and sh-PLK4, empty vector and PLK4 overexpression) and packaging plasmids (VSVG and ΔR) were transfected into HEK293T cells supplemented with PEI (Polysciences, Warrington, USA). SW480 and HCT8 cells were used for transfection. Polybrene (Solarbio, Beijing, China) was applied as the transfection reagent. Stably transfected SW480 and HCT8 cell lines were obtained under puromycin (Gibco, New York, USA) selection.

### Immunohistochemical staining

Immunohistochemistry (IHC) was applied to assess the protein levels of PLK4, Ki67, p-ERK and p-p38 in paraffin-embedded samples of CRC tissue according to previously described methods^17^. An anti-PLK4 antibody was used at the concentration of 1:200 (Abcam, Cambridge, UK), and anti-Ki67, p-ERK, p-p38 antibody was used at the concentration of 1:500, 1:800, 1:500, respectively (Cell Signaling Technology, Danvers, USA). The percentage of PLK4-positive cells was evaluated from 0 to 3 (0, no positive cells; 1, < 30% positive cells; 2, 30-60% positive cells; and 3, 60%-100% positive cells). The PLK4 staining intensity was scored across a range of four grades (0, no positive staining; 1, weakly positive staining; 2, moderately positive staining; and 3, strongly positive staining). Finally, the IHC staining intensity and percentage scores were multiplied using a specific formula to obtain an intermediate score, which was then used to calculate the final IHC score.

### Western Blotting and antibodies

The following antibodies were used: anti-PLK4 (Rabbit, 1:1,000), and anti-PCNA (Mouse, 1:1,000) from Abcam (Cambridge, the United Kingdom); anti-p-p38 (Rabbit, 1:1,000), anti-p38 (Rabbit, 1:1,000), anti-p-ERK (Rabbit, 1:4,000), anti-ERK (Rabbit, 1:1,000), anti-Beclin1 (Rabbit, 1:1000), anti-Atg5 (Rabbit, 1:1000), anti-LC3B (Rabbit, 1:1000) and anti-SQSTM1/p62 (Rabbit, 1:1000) from Cell Signaling Technology (Danvers, USA); anti-caspase 3 (Rabbit, 1:500) from Proteintech (Chicago, IL, USA); anti-Bax (Rabbit, 1:1000) and anti-GAPDH (Mouse, 1:1000), anti-CDK4 (Mouse, 1:1000), anti-CDK6 (Rabbit, 1:1000) from Bioss (Beijing, China). The steps for WB were based on previously described methods^17^.

### RNA extraction, cDNA synthesis, and qRT-PCR

Total RNA was separated from adherent cells with TRIzol reagent (Ambion, Texas, USA). According to a qRT-PCR kit (Takara, Tokyo, Japan), cDNA was synthesized by reverse transcription of the isolated RNA. The amplification reaction was executed with designed primers based on the manufacturer's instructions (Takara). The primer sequences are shown in Table 1.

### CCK-8 assay

According to the protocol of the CCK-8 assay, sh-PLK4 and sh-control transfected SW480 and HCT8 cells were plated in a 96-well plate at a density of 1-4 × 10^3^ cells per well. Six parallel wells were used for each independent group. Ten microliters of CCK-8 (Dojindo Laboratories, Kyushu, Japan) reagent were mixed in each well and incubated with the cells for 4 hours at 37°C in 5% CO_2_. The optical density (OD) value at 450 nm was evaluated with an enzyme-labeling instrument. Cell viability was assessed on days 0, 1, 2 and 3.

### Colony formation assay

For cell proliferation analysis, sh-PLK4 and sh-control transfected CRC cells were seeded in 6-well plates (500-1,000 cells/well) and incubated at 37°C in 5% CO_2_ for 1-2 weeks. Once colonies were evident, the cells were stained with crystal violet and photographed with a digital camera.

### Apoptosis and Cell cycle assay

CRC cells were washed with ice-cold PBS and then suspended in 200 μL of 1× binding buffer, followed by staining for 15 minutes in the dark with Annexin V-PE and 7-AAD (eBioscience, California, USA). Based on the cell number, 400-800 μL of 1× binding buffer was added and mixed, and then the cells were evaluated by BD FCSCanto II (Becton, Dickinson and Company, New Jersey, USA). The percentage of apoptotic cells was assessed with flow cytometric analysis. CRC cells were digested for cell cycle assay, mixed in 95% ethanol and incubated at 4°C overnight. Following centrifugation and washing, the cells were incubated with 500 μL of propidium iodide (PI; BD Biosciences, New Jersey, USA) and stained in the dark for 15 minutes. The cell samples were assessed on a FACS Aria flow cytometer (BD) with CellQuest software. The percentage of apoptotic cells and the cell cycle results were evaluated with FlowJo software.

### EDU assay

A BeyoClick^TM^ EdU assay kit (Beyotime, Shanghai, China) was adopted to inquire the cell proliferation ability. Cells were seeded into plates with a density of 1 × 10^5^ cells each well. Then cells incubated with 10 μM EDU buffer at 37 °C for 2 hours were fixed with 4% paraformaldehyde for 15 minutes and permeabilized with 0.3% Triton X-100 for 10 minutes. EDU solution was added into culture followed by the staining of nuclei with Hoechst. The results were visualized by a fluorescence microscope.

### Scratch assay

CRC cells were harvested at a concentration of 1.5-2 × 10^6^ cells per well in 6-well plates. The next day, an equal-width scratch was made through the CRC cell monolayer in the 6-well plates by using a 2.5-μL pipette tip. Then, the cells were washed with PBS two or three times, and 2% FBS was mixed into the DMEM. The healing of the scratch was recorded by assessing the reductions in the distance between the edges measured at a suitable time (3, 6, 9, 12, or 24 hours) and comparing those distances with the average distance measured at six stochastic locations at 0 hour. Images were captured at 0 and 24 h with a microscope at 10× magnification. Data are shown as the mean ± SD.

### Migration and invasion assays

For the migration and invasion assays, DMEM supplemented with 20% FBS was loaded into the lower chamber of a transwell system. A total of 1 × 10^5^ cells in 2% FBS DMEM were plated on an 8-μm polyvinyl pyrrolidone-free polycarbonate filter membrane (Corning) for the migration assay. For the invasion assay, Matrigel-coated transwell chambers were incubated for more than 1 hour at 37°C. After incubating for approximately 24 h at 37°C in 5% CO_2_, the migrated or invaded cells on the bottom were fixed with 4% paraformaldehyde (PFA) for 20 minutes and stained.

### β-galactosidase staining for cellular senescence

Cellular senescence was evaluated by β-galactosidase (β-gal) staining using the Senescence β-Galactosidase Staining Kit (Solarbio, Beijing, China) following the manufacturer's instructions. Briefly, cells were fixed with 2% formaldehyde and 0.2% glutaraldehyde for 10 minutes at room temperature, and then incubated with β-gal staining solution overnight at 37°C in a dry incubator without CO_2_. Cells with blue staining in the cytoplasm were considered to be senescent cells.

### The Cancer Genome Atlas and Gene Expression Omnibus datasets

Raw data from The Cancer Genome Atlas (TCGA; National Cancer Institute) and Gene Expression Omnibus (GEO; NCBI) databases for CRC were downloaded from the official database websites. Then, the original data were normalized with R Studio. We evaluated the expression of PLK4 in tumor, adjacent normal tissue and polyp tissue samples from the TCGA, GSE41657 and GSE41258 mRNA expression datasets.

### Gene set enrichment analysis

Gene set enrichment analysis (GSEA) was used to evaluate whether the *PLK4* mRNA level was correlated with CRC biological features and signaling pathways, including proliferation, cell cycling, tumor metastasis, invasiveness and patient survival, based on of the GSE32323 dataset for CRC evaluated with GSEA 4.0.0 (The Broad Institute of MIT and Harvard).

### Autophagic flux counting

The cells were transfected with stubRFP-sensGFP-LC3 lentivirus, which was obtained from GENE-CHEM and the manufacturer's protocol was followed. Following transfection with mRFPGFP-LC3, yellow labeling (mRFP and GFP) and red labeling (mRFP only) were used to label autophagosomes and autolysosomes, respectively. Finally, fluorescence microscopy was used to visualize the cells.

### Transmission electron microscopy (TEM) imaging of autophagosomes and autolysosomes

Cells were fixed with 2.5% glutaraldehyde in 0.1 M cacodylate buffer (pH 7.4) at 4°C for 2 hours, followed by post-fixation with 1% osmium tetroxide for 1 hour at room temperature. The samples were dehydrated with a series of graded ethanol solutions and embedded in epoxy resin. Ultrathin sections (70 nm) were cut using an ultramicrotome and mounted on copper grids. The sections were stained with uranyl acetate and lead citrate and examined under a transmission electron microscope (HITACHI, Tokyo, Japan) and the images were captured by Wuhan servicebio technology CO., LTD and analyzed using image processing software (ImageJ, National Institutes of Health, Bethesda, MD, USA). Autophagosomes were identified as double-membrane vesicles containing undigested cytoplasmic material, while autolysosomes were characterized by single-membrane vesicles containing partially digested cytoplasmic material and electron-dense lysosomal contents.

### *In vivo* experiments

Four to five-week-old nude mice (SPF Biotechnology Co., Ltd., Beijing, China) were acquired for xenograft animal assays (n = 8 per group). sh-control/HCT8 and sh-PLK4/HCT8 cells were prepared, and 5 × 10^6^ cells in 100 μL of PBS were injected subcutaneously. Tumor volume was measured every three days using a Vernier caliper. To establish lung metastasis models, prepared sh-control/HCT8 and sh-PLK4/HCT8 cells (5 mice/group, 2 × 10^6^/mL; 100 μL per mouse) were injected into the tail vein. All anaesthetic operations were performed by intraperitoneal injection with 1 mg/mL barbitone. After six weeks, the mice were sacrificed, and the lungs were harvested. After a pneumonectomy procedure, it is commonly recommended to utilize picric acid staining to help visualize and identify any abnormalities or changes in the lung tissue. Followed by a decolorizing step to remove excess picric acid, the tissues were used for hematoxylin and eosin (H&E) staining.

### Statistical analyses

Clinical data were processed and assessed by using SPSS 24.0 for Windows (SPSS Inc., Chicago, IL). The univariate Kaplan-Meier method and multivariate Cox method were used to evaluate the independent risk factors and survival curves of CRC patients. Spearman correlation analysis was used to examine the correlations between the PLK4 staining score and clinicopathological factors.

## Results

### Knockdown of PLK4 promotes dormancy of CRC cells *in vitro*

**Figure 1:**
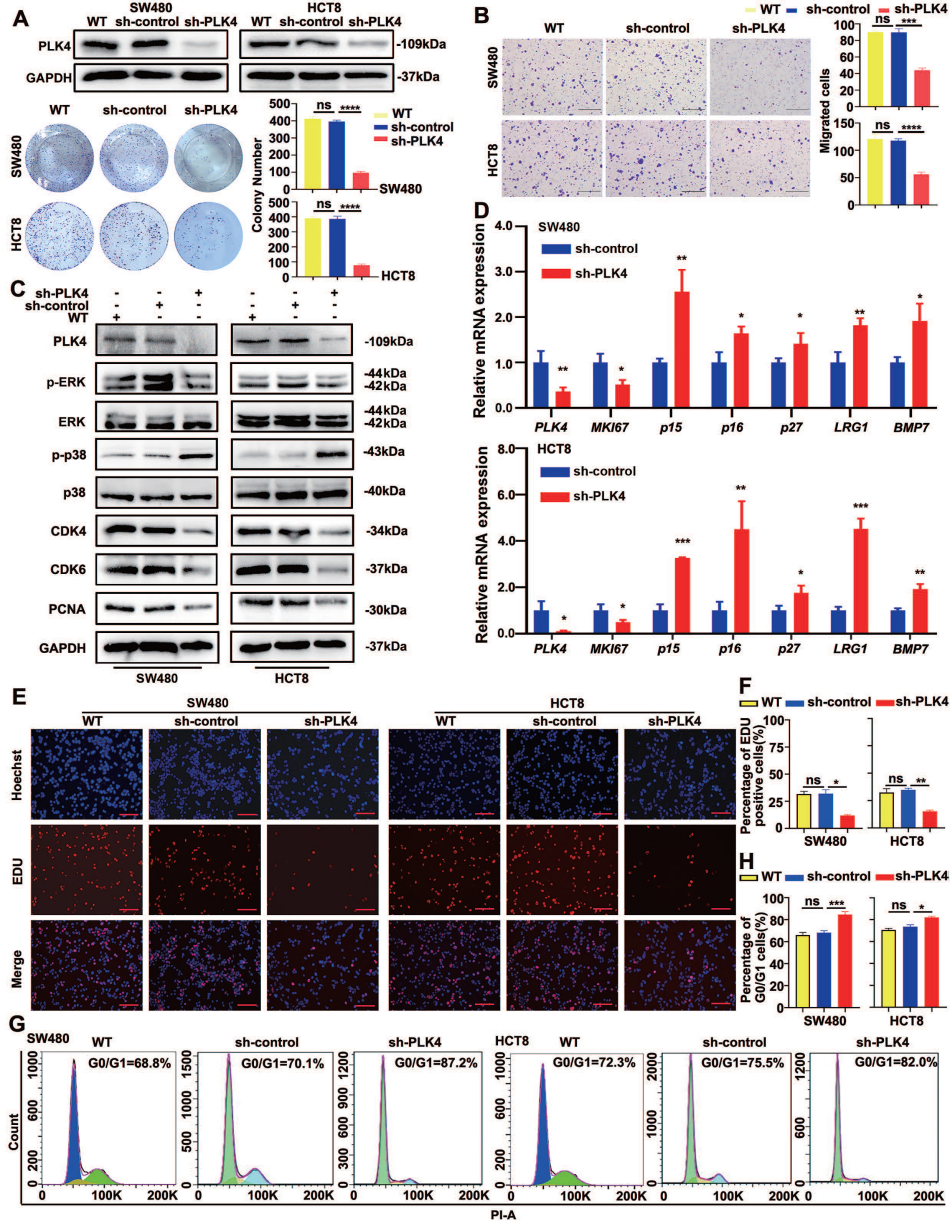
** Knockdown of PLK4 promotes dormancy of CRC *in vitro*.** A. The wildtype (WT), construction of PLK4-control (sh-control) and PLK4-knockdown (sh-PLK4) SW480 and HCT8 CRC cell lines and clone formation assay revealing the proliferative abilities of CRC cells in the three groups. B. Transwell assays comparing cell invasion differences between the three groups of SW480 and HCT8 cells (scale bar, 1.0 mm). C. The protein levels of PLK4, cell proliferation and cell cycle markers as assessed by WB. D. qRT-PCR assay evaluating signaling molecules favoring the acquisition of a dormant phenotype in the sh-control and sh-PLK4 groups of SW480 and HCT8 cells. E-F. Immunofluorescence assay revealing that downregulation of PLK4 expression induced proliferative transformation in WT, sh-control and sh-PLK4 groups of SW480 and HCT8 cells. Representative EDU staining images (E) and EDU-positive cell rates (F) are shown (scale bar: 50 μm). G-H. Cell cycle assay revealing the difference in the G0/G1 cell cycle proportion between the WT, sh-control and sh-PLK4 groups of CRC cell lines. Representative cell cycle images (G) and percentage of G0/G1 cells (H) are shown. Data were presented as mean ± SEM. n = 3. ns: *p* > 0.05, * *p* < 0.05, ** *p* < 0.01, *** *p* < 0.001, **** *p* < 0.0001.

Stable knockdown of PLK4 and the control cells (sh-PLK4 and sh-control) were carried out in SW480 and HCT8 cells respectively to investigate the potential biological function of PLK4 in CRC progression. The efficiency of PLK4 knockdown were verified by WB and qRT-PCR, functional validation *in vitro* including clone formation and the CCK-8 assays were applied to detect cell proliferation abilities, it turned out that PLK4 deficiency considerably restricted the proliferation of CRC cells, however, there was no significant difference observed in the proliferation of the wild-type and control group cells (Figure 1A and Figure S1A&B). Moreover, cell migration and invasion were substantially impaired by endogenous suppression of PLK4 by using wound-healing, chemotaxis and transwell assays (Figure 1B and Figure S1D&E).

GSEA was then applied to derive enrichment scores for CRC samples with high or low PLK4 expression in the GSE32323 dataset. It was verified in the dataset that significant positive correlations were observed between highly expressed *PLK4* and enriched proliferation- and cell cycle-related genes, such as *MKI67*,* PCNA* and *CCND1* (Figure S1C). As mentioned above, PLK4 expression is a critical factor for evaluating tumor proliferation, thus we intended to explore the relationship between PLK4 expression and CRC dormancy.

It is noteworthy that the p-p38^high^/p-ERK^low^ signaling ratio is already widely used in the identification of tumor cell dormancy^18^. CDK4 and CDK6 are critical mediators of the cellular transition from the G1 phase to the S phase of the cell cycle, when DNA synthesis occurs, by binding to the D-type cyclins (including cyclin D1, cyclin D2 and cyclin D3)^19^, ^20^. CDK4 is the most important protein kinase in the G1 phase of cells and can synergize with CDK6, which makes it a potential marker for dormancy activation^21^, ^22^. In accordance with previous studies, WB analysis showed a prominently increased p-p38 level and a decreased p-ERK level caused by PLK4 depletion, and PLK4-depleted dormant-phenotype CRC cells alleviated the expression of important indicators of cell proliferation and cell cycle status, such as PCNA, CDK4 and CDK6 (Figure 1C). Simultaneously, signaling molecules favoring the acquisition of a dormant phenotype, such as* p15, p16, p27, MKI67, LRG1* and* BMP7*, were further characterized by a qRT-PCR assay. Consistently, upregulated* p15, p16, p27, LRG1* and* BMP7* mRNA expression in CRC cell lines was observed with PLK4 deficiency, whereas *MKI67* mRNA expression exhibited the opposite trend (Figure 1D).

Meanwhile, the EDU assay was utilized to assess the proliferation capacity of CRC cells and the cell cycle was evaluated in SW480 and HCT8 cells by flow cytometry. We found that downregulation of PLK4 expression in CRC cells directly resulted in a reduced proliferation capacity of the cells (Figure 1E&F) and substantial G0/G1 cell cycle arrest, characteristics of dormancy (Figure 1G&H). It is well-known that both quiescent and senescent cells exhibit a G0/G1 arrest state. Cellular senescence is characterized by cell-cycle arrest in the G1 or possibly G2 phase, which prevents the proliferation of damaged cells^23^, ^24^. On the other hand, cancer cell dormancy is a process that allows cycling cells to reversibly cease proliferation and typically occurs in the G0 phase^25^. Senescence-associated beta-galactosidase (SA-beta-gal or SA-β- gal) is a widely used biomarker for senescent and aging cells^26^. However, when we used SA-β-gal staining to assess senescence, we found no discernible contrast in blue staining between sh-control and sh-PLK4 in CRC cells, suggesting that sh-PLK4 cells undergo dormancy, rather than senescence (Figure S1F). The above results suggested a regulatory role for PLK4 in regulating tumor cell dormancy in CRC.

### Upregulated PLK4 expression facilitates CRC cells malignancy potential

To further characterize the role of PLK4 in CRC progression, we next performed GSEA based on the mRNA data of CRC samples in a GEO dataset (GSE32323). The results showed that high PLK4 expression was remarkably associated with a series of malignant behaviors related to an unfavorable prognosis for CRC (Figure S2A-F). Briefly, high expression of PLK4 was observed in CRC samples (*p* = 0.010, Figure S2A). Importantly, PLK4 expression in CRC samples was positively associated with proliferation (*p* < 0.0001, Figure S2B) and higher expression of PLK4 usually exhibited an activated cell cycle (*p*=0.040, Figure S2C), enhanced invasiveness (*p* = 0.006, Figure S2D) as well as metastasis (*p* < 0.0001, Figure S2E). However, the difference of PLK4 expression for recurrence was not statistically significant (*p* = 0.076, Figure S2F). Overall, increased expression of PLK4 was found to be a critical risk factor for the development of CRC, suggesting that PLK4 does play a vital role in CRC progression.

**Figure 2:**
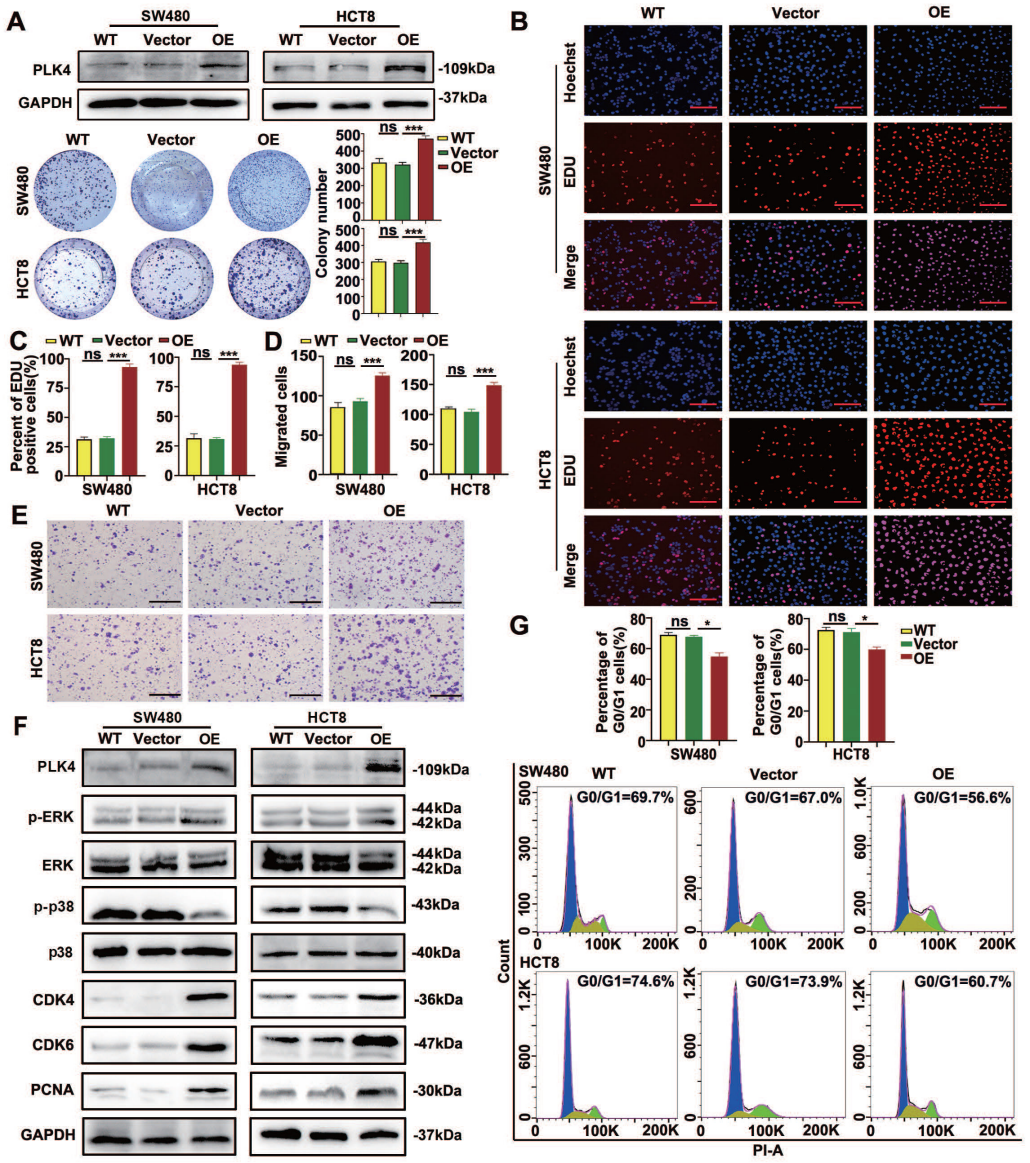
** Upregulated PLK4 expression facilitates CRC cells malignancy potential.** A. The construction of empty-vector and PLK4 overexpression (PLK4 OE) SW480 and HCT8 CRC cell lines. The PLK4 protein level assessed by western blotting (up) and the clone formation ability (down) are shown among WT, Vector and OE groups. B-C. EDU assays revealing the proliferative abilities of SW480 and HCT8 cells in the WT, Vector and PLK4 OE groups. Representative EDU staining images (B) and EDU-positive cell rates (C) are shown (scale bar, 1.0 mm). D-E. Chemotaxis assay comparing cell migration differences among the WT, Vector and PLK4 OE groups of CRC cells. The migrated cells (D) and representative migrated cell images (E) are shown. F. The protein levels of PLK4 and dormancy-associated markers as assessed by western blotting. G. The percentage of G0/G1 phase cells (up) and representative cell cycle images (down) in the WT, Vector and PLK4 OE groups. Data were presented as mean ± SEM. n = 3-4. ns: *p* > 0.05, * *p* < 0.05, *** *p* < 0.001.

At the same time, we constructed the overexpression of PLK4 (OE) and empty vector (Vector) in CRC cells and verified the efficiency of PLK4 overexpression by WB and qRT-PCR (Figure 2A and Figure S1A). Based on clone formation and EDU assays, PLK4 overexpression considerably enhanced the proliferation of CRC cells (Figure 2A-C). The movement capability and invasion ability of tumor cells were stronger when PLK4 was overexpressed by using chemotaxis and transwell assays (Figure 2D&E and Figure S1E). WB analysis led to an opposite effect on dormancy-associated proteins, showed a decreased p-p38 level and an increased p-ERK level caused by PLK4 overexpression, accompanied by the increased expression of PCNA, CDK4 and CDK6 (Figure 2F). Flow cytometric analysis demonstrated that overexpression of PLK4 can affect the distribution of cells in the cell cycle. Specifically, the mean percentage of cells in the G0/G1 phase decreased from 68.2% to 56.5% in SW480 cells and from 75% to 61.8% in HCT8 cells (Figure 2G). In summary, the CRC cell biological characteristics changed by PLK4 expression strongly suggest a tumor promoting function for PLK4 in CRC progression.

### Low expression of PLK4 is critical for evaluating CRC dormancy in clinical samples

**Figure 3:**
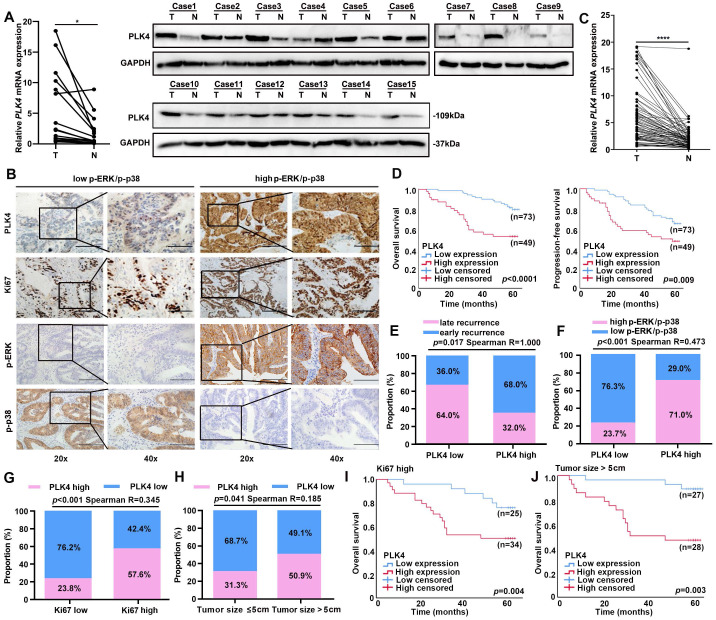
** Low expression of PLK4 is important for evaluating tumor dormancy in clinical level.** A. The mRNA and protein levels in 15 pairs of CRC tissues and adjacent nonmalignant tissues by qRT-PCR (left) and western blotting assay (right) respectively. B. IHC assay revealing PLK4 as a key node between dormancy and proliferation in 122 CRC samples. The representative staining images of PLK4, Ki67, p-ERK and p-P38 are shown. (scale bar, 100 μm; magnification, 200 × and 400 ×). C. The qRT-PCR assay showed differences in PLK4 mRNA levels in additional 75 pairs of CRC tissues and adjacent nonmalignant tissues derived from 122 CRC patients of (B). D. Kaplan-Meier survival curve validating the correlation between the PLK4 expression level and CRC patients' OS and PFS. E. Correlation analysis between PLK4 expression and different recurrence types. F-G. Correlation analysis between PLK4 expression and the level of p-ERK/p-p38 and Ki67. H. Correlation analysis between the PLK4 IHC score and clinicopathological factor tumor size in the CRC cohort. I-J. Kaplan-Meier survival curve showing the correlation between the PLK4 IHC score and OS of the high-risk subgroup in the CRC cohort. T: Tumor, N: adjacent non-tumor tissue, Data were presented as mean ± SEM. * *p* < 0.05, **** *p* < 0.0001.

The expression level pattern of PLK4 between tumor and adjacent non-tumor tissues was first analyzed with TCGA Pan-cancer datasets. Remarkably, we found that PLK4 expression was dramatically enhanced in the tumor tissues compared with the adjacent non-tumor tissues in most cancer types, including CRC (Figure S2G). Notably, the above results were further confirmed by comparing the expression of PLK4 in fifty paired CRC and adjacent non-tumor tissues in the GEO CRC datasets (GSE41657 and GSE41258) (Figure S2H). To validate the expression of PLK4, we collected 15 CRC patient-matched fresh tumor and adjacent non-tumor tissue. Consistently, both the qRT-PCR analysis and WB analysis of the 15 paired CRC and adjacent non-tumor tissue specimens indicated that 14 out of the 15 pairs of samples had higher levels of PLK4 expression in the tumor tissues (Figure 3A). Collectively, these results suggested that PLK4 expression was upregulated in CRC tissues.

To further characterize PLK4 in clinical data, a cohort of 122 patients with CRC was collected. The expression pattern of PLK4 was detected by IHC. Next, the Kaplan-Meier method and a Cox regression model were applied to explore the correlation between PLK4 and the prognosis of CRC patients. Multivariate analysis showed that increased expression of PLK4 was one of the two independent risk factors for the prognosis of CRC (Table 2). Moreover, the IHC score cutoff value is used to distinguish between PLK4 low and high expression and we divided these patients into two groups based on their IHC scores for PLK4 (Figure 3B). We obtained 75 paired CRC patient-matched tumor and adjacent non-tumor tissue specimens among the 122 patients, these specimens further confirmed that PLK4 expression was significantly higher in CRC tissues (Figure 3C). Additionally, survival analysis revealed that patients with low PLK4 expression had a significantly higher 5-year overall survival (OS) and progression-free survival (PFS) rate compared to those with high PLK4 expression, which demonstrated a crucial role for PLK4 in CRC progression (Figure 3D).

Previous literature has shown that the majority of CRC recurrence is reported within 2 years after radical treatment^27^. This cohort of CRCs was enrolled and divided into early (< 2 years) and late (≥ 2 years) recurrence groups. Remarkably, we found that the low PLK4 expression was highly consistent with CRC late recurrence, the percentage of patients with low PLK4 expression in the group with p-p38^high^/ p-ERK^low^ signaling ratio was significantly higher than that in the corresponding group with p-ERK^high^/p-p38^low^ signaling ratio (Figure 3E&F). Correspondingly, the percentage of patients with high PLK4 expression among the high-risk subgroups of patients with larger tumor size (> 5 cm) and elevated Ki67 expression was significantly higher than that in the corresponding low-risk subgroups (Figure 3G&H). And in the high-risk subgroups mentioned above, the CRC patients with low expression levels of PLK4 exhibited a better prognosis than those with high PLK4 expression levels (Figure 3I&J). These results indicate that PLK4 serves as a key node between dormancy and proliferation in CRC and PLK4 low expression is critical for evaluating CRC dormancy in clinical samples.

Considering the important role of PLK4 in CRC progression, we further analyzed the correlations between PLK4 and various clinicopathological factors in our CRC cohort. Notably, we found that the expression of PLK4 was markedly correlated with Ki67 expression level (*p <* 0.001), tumor size (*p* = 0.041), TMN stage (*p* = 0.002), CEA (*p* = 0.016), lymph node metastasis status (*p* = 0.012) and tumor capsule status (*p* = 0.001) (Table 3). Remarkably, the percentage of patients with high PLK4 expression among the high-risk subgroups of patients with positive lymph node metastasis status, ruptured-capsule tumors elevated CEA (≥ 5 ng/ml) or advanced TMN stage (stage III & IV) was significantly higher than that in the corresponding low-risk subgroups (Figure S2I-L). Consistently, in the high-risk subgroups above, the CRC patients with low levels of PLK4 expression exhibited a better prognosis than those with high PLK4 expression levels (Figure S2M-P). Thus, these findings indicate that PLK4 could serve as a promising biomarker for predicting the prognosis of CRC and low expression of PLK4 is a critical factor for evaluating tumor dormancy.

### PLK4 downregulation induced autophagy was sufficient to maintain CRC cell dormancy

**Figure 4:**
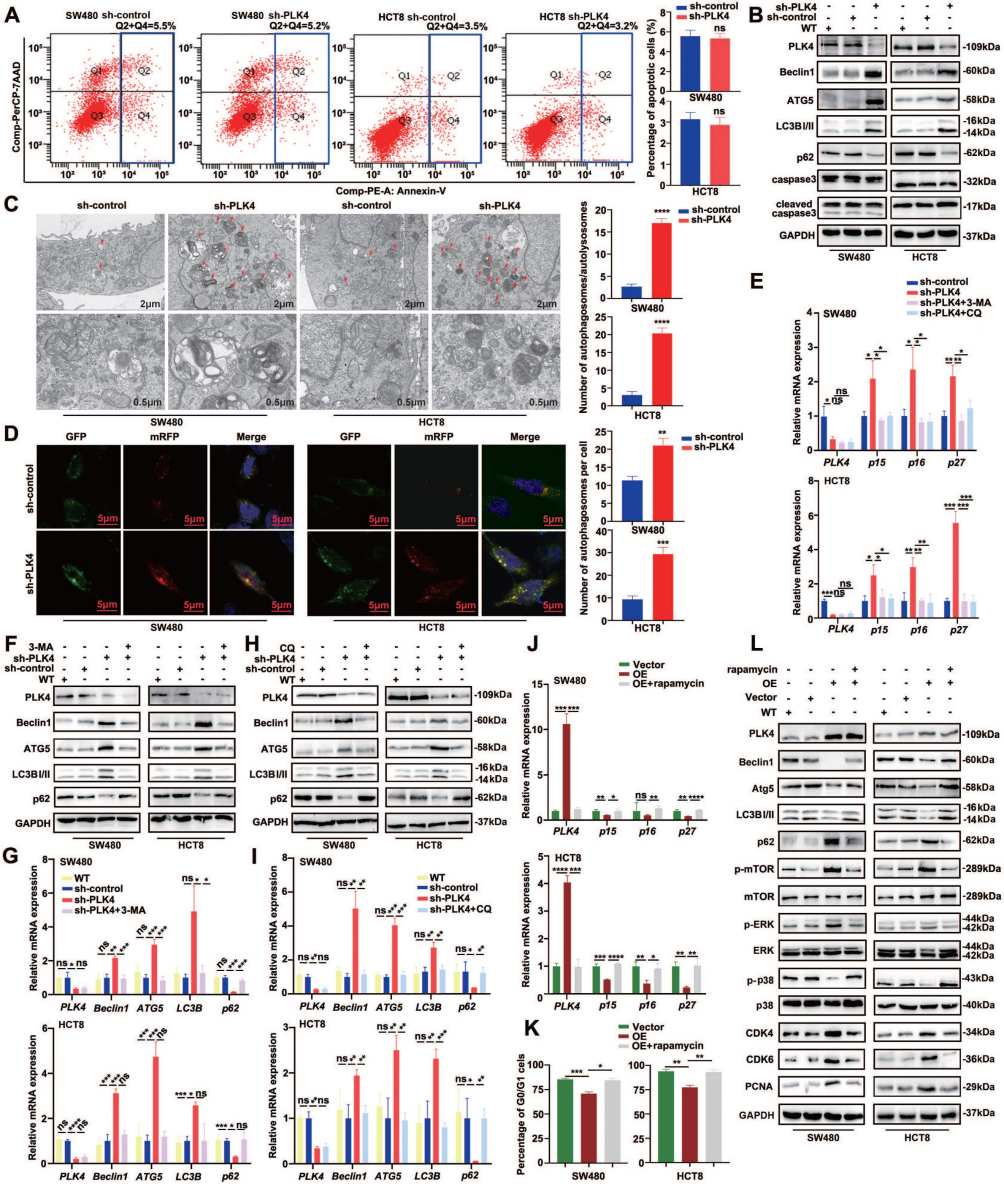
** PLK4 downregulation induced autophagy was sufficient to initiate CRC cell dormancy.** A. Cell apoptosis assay revealing the proportions of apoptotic SW480 and HCT8 cells in the sh-control and sh-PLK4 groups. B. Western blotting to detect the effect of PLK4 on the expression levels of autophagy-related proteins including Beclin1, ATG5, LC3BI/II, and p62 and apoptosis-related markers including caspase3 and cleaved caspase3. C. Representative transmission electron microscopy images of sh-control and sh-PLK4 cells (left). Chromatin condensation and nuclear fragmentation are indicated by arrows (scale bar, 2μm (up) and 0.5μm (down)). The number of autophagosomes/autolysosomes was quantified (right). D. SW480 sh-control, sh-PLK4 and HCT8 sh-control, sh-PLK4 transfected with GFP-mRFP-LC3 lentivirus. Fluorescence microscopy was used to acquire images of yellow dots (autophagosomes) or red-only dots (autolysosomes), and the average number of autophagosomes in the merged images per cell was quantified (scale bar, 5 μm). E. The mRNA levels of *PLK4*, *p15*, *p16* and *p27* in sh-control and sh-PLK4 groups with or without 3-MA or CQ. F-I. The protein levels (F&H) and mRNA levels (G&I) of PLK4 and autophagy-associated elements assessed by western blotting in WT, sh-control and sh-PLK4 cells with or without 3-MA or CQ. J. The mRNA levels of *PLK4*, *p15*, *p16* and *p27* in Vector and PLK4 OE groups with or without rapamycin. K. Cell cycle assay revealing the G0/G1 difference in WT, Vector and PLK4 OE groups with or without rapamycin. L. The effect of autophagy inducer on autophagy and dormancy markers verified via western blotting. Data were presented as mean ± SEM. n = 3-4. ns: *p* > 0.05, * *p* < 0.05, ** *p* < 0.01, *** *p* < 0.001, **** *p* < 0.0001.

No remarkable difference in apoptosis or necrosis was observed between the sh-control and sh-PLK4 groups for these two cell lines (Figure 4A&B). How to induce dormant cells apoptosis and eradicate them might be used as a therapeutic target in future. Autophagy may serve as upstream regulation mechanism of apoptosis and it was reported to be highly active in dormant cancer cells, therefore, autophagy plays an important role in the initiation and maintenance of tumor cell dormant state^11^, ^28^. Intriguingly, WB revealed that the expression of Beclin1, Atg5 and LC3B-II were strongly increased while p62 was decreased in the cell lines with PLK4 knockdown. To further assess the effect of PLK4 on autophagy, we utilized transmission electronic microscopy (TEM) to evaluate the quantity of autophagosomes/autolysosomes in SW480 and HCT8 cells with or without PLK4 downregulation. TEM data revealed that numbers of autophagosomes/autolysosomes increased in sh-PLK4 cells compared with sh-control cells (Figure 4C). Furthermore, we employed the mRFP-GFP-LC3B double-fluorescence system to assess autophagosomes/autolysosomes in different cell lines. Our data revealed that the numbers of yellow dots (autophagosomes) and red-only dots (autolysosomes) significantly increased in the cell lines with PLK4 downregulation (Figure 4D). In accordance with the previous studies, upregulated *p15, p16* and* p27* mRNA expression in CRC cell lines was observed with PLK4 deficiency, and these results were reversed when PLK4-knockdown cells were treated with autophagy inhibitor 3-MA or CQ (Figure 4E).

To investigate the relationship between PLK4 knockdown and autophagy, we examined several autophagy-associated elements. we observed a significant increase in the expression of Beclin1, Atg5, and LC3B-II, while the expression of p62 was reduced in the cell lines with PLK4 deficiency. This finding was further confirmed by both WB and qRT-PCR analyses. In contrast, when PLK4-knockdown cells were treated with the autophagy inhibitors 3-MA (Figure 4F&G) or CQ (Figure 4H&I), we observed the opposite trend in the expression of these autophagy-related elements. These results suggested that PLK4 knockdown induced autophagy in the cell lines, and the inhibition of autophagy by 3-MA or CQ could reverse these effects. Overall, our findings support the notion that PLK4 plays a crucial role in regulating autophagy.

The mechanistic Target of Rapamycin (mTOR), a serine/threonine kinase, is a master regulator of cellular growth and proliferation^29^. The activity of autophagy is tightly regulated by protein complexes that contain the mTOR kinase^30^. mTOR is so named because it is inhibited by rapamycin and is a well-known key regulator of autophagy which is generally suppressed by the activation of mTOR in various systems^31^, ^32^. To further investigate whether autophagy participated in PLK4-induced effects on dormancy, we conducted the autophagy inducer, rapamycin, in the cell lines with PLK4 overexpression. Downregulated* p15, p16* and *p27* mRNA expression in CRC cell lines was observed with PLK4 overexpression, whereas the mRNA expression exhibited the opposite trend when cells were treated with rapamycin (Figure 4J). Furthermore, PLK4 overexpression cells treated with rapamycin exhibited a more remarkable G0/G1 arrest (Figure 4K & Figure S3A). Accompanied by the parallel results from WB, compared with Vector group, upregulated PLK4 expression caused an increase in the expression of p-mTOR, CDK4, CDK6, and PCNA, to some extent, caused dormancy-associated protein expression level changes. In contrast, treatment of PLK4-upregulated CRC cells with rapamycin resulted in a significant increase in Beclin1, Atg5 and LC3B-II, as well as p-p38, and a marked reduction in the expression of p-mTOR, CDK4, CDK6, PCNA, and p62 (Figure 4L). Collectively, these data implied that PLK4 downregulation induced autophagy was sufficient to maintain tumor cell dormancy.

### Autophagy inhibition facilitates dormant cells apoptosis

**Figure 5:**
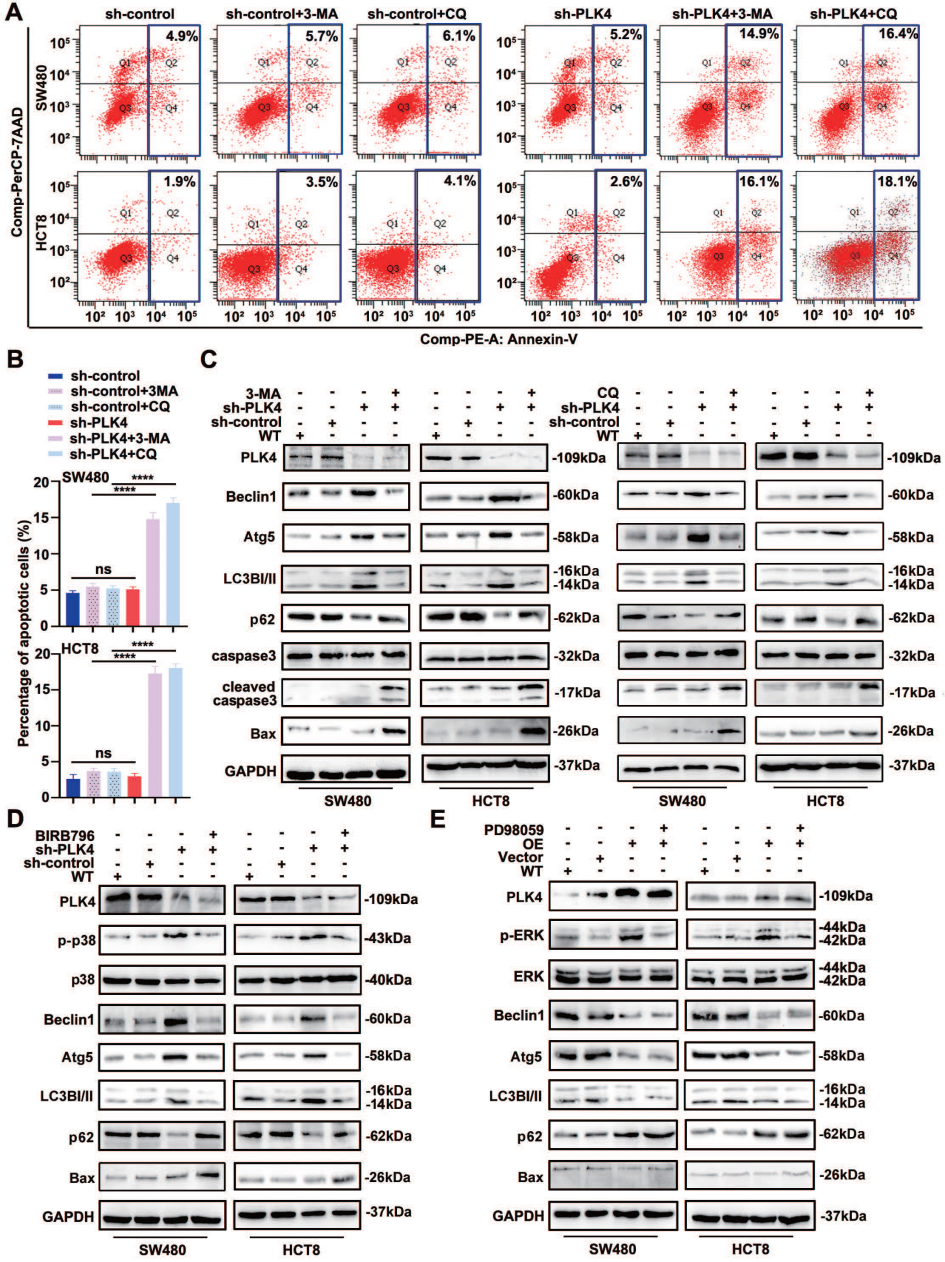
** Autophagy inhibition facilitates dormant cells apoptosis.** A-B. Apoptotic rates were measured in sh-control and sh-PLK4 groups with or without 3-MA or CQ and quantification of apoptotic rate measurement. C. Western blotting analysis of apoptosis and autophagy-related protein levels in WT, sh-control and sh-PLK4 cells with or without 3-MA or CQ. D. Western blotting analysis of the protein levels of p-p38, p38, autophagy-related markers and apoptosis marker in WT, sh-control and sh-PLK4 cells pretreated with or without BIRB796. E. Western blotting analysis of the protein levels of p-ERK, ERK, autophagy-related marker and apoptosis marker in WT, Vector and PLK4 OE cells pretreated with or without PD98059. Data were presented as mean ± SEM. n = 3-4. ns: *p* > 0.05, **** *p* < 0.0001.

Considering that autophagy is a critical survival process activated and maintained tumor dormancy and dormant tumor cells undergo cell apoptosis following autophagy inhibition^33^. In accordance with the results above, further analysis identified that PLK4-knockdown cells were treated with autophagy inhibitor 3-MA or CQ, the apoptosis rate increased while autophagy inhibitor caused little impact on apoptosis rate in PLK4-control cells (Figure 5A-C). Actually, PLK4 overexpressed cells treated with rapamycin prominently alleviated the expression of p-p38 (Figure 4J) and the expression of PLK4 mainly increased the protein level of p-p38 and the level of p-ERK was relatively less influenced (Figure 1C&2F). Given p38/MAPK signaling pathway associated with autophagy, we treated PLK4-knockdown cells with 30 μM BIRB796, which was p38 inhibitor, to examine the relationship of the MAPK signaling pathway with apoptosis and autophagy.

As expected, we confirmed that the BIRB796-mediated inhibition of p-p38 significantly reduced the levels of the autophagy-related proteins Atg5, Beclin1, LC3B-II, while increased p62 and the levels of the apoptosis-related proteins Bax in PLK4-knockdown cells (Figure 5D). Additionally, we investigated the mRNA levels of autophagy-related proteins upon PLK4 knockdown and treatment with the p38 inhibitor BIRB796. The BIRB796-mediated inhibition of p-p38 significantly reduced the levels of *Atg5*, *Beclin1*, and *LC3B*, while increasing *p62* levels, which was consistent with the protein data (Figure S3B). Then we treated PLK4-overexpression cells with PD98059, which was MEK/ERK inhibitor. p-ERK expression significantly increased in PLK4-overexpression cells but effectively suppressed upon treatment with PD98059, while caused little effect on Beclin1 or Atg5. Though the expression level of selective autophagy receptor LC3B-II slightly changed, apoptosis-associated protein expression levels did not significantly change (Figure 5E). This signified that the percentage of apoptotic dormant tumor cells increased significantly following autophagy inhibition and PLK4 downregulation induced autophagy regulated cell dormancy mainly through the p38 MAPK signaling pathway.

### PLK4 downregulation in CRC cells facilitates autophagy-induced dormancy *in vivo*

**Figure 6:**
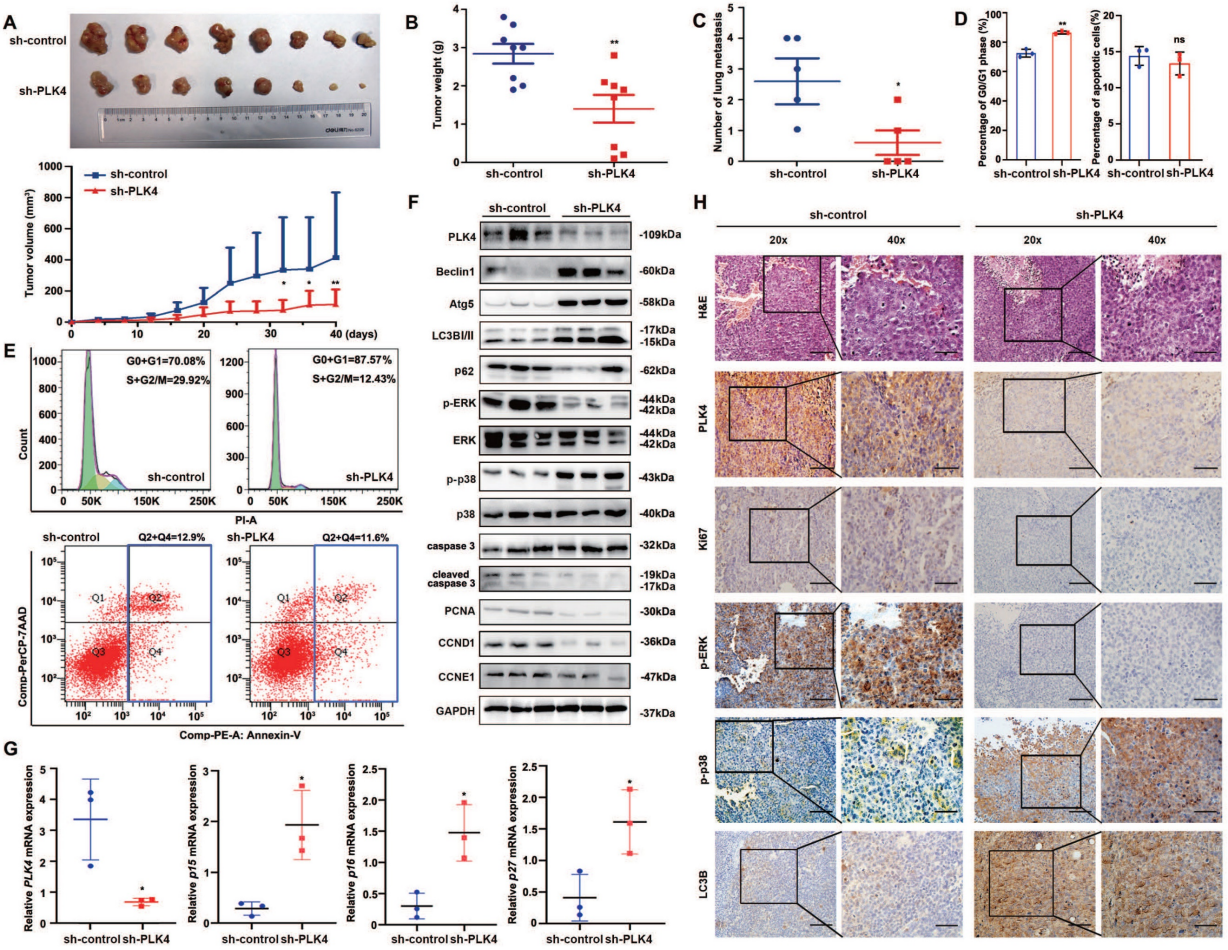
** PLK4 downregulation facilitates autophagy-induced dormancy *in vivo.*** A-E. The primary tumor growth rates and volumes (A), tumor weights (B), numbers of lung metastasis nodules (C), the cell cycle and apoptosis differences (D) and representative flow cytometry images of cell cycle and apoptosis (E) in the sh-control and sh-PLK4 groups. F. The protein levels of PLK4, autophagy-associated proteins, cell proliferation and cell cycle markers in the xenograft model, as assessed by WB. G. The mRNA levels of *PLK4*, *p15, p16* and *p27* detected by qRT-PCR in the sh-control and sh-PLK4 tumor tissues. H. Verification of PLK4 and cell proliferation, cell cycle and autophagy markers in the xenograft model, as assessed by IHC (scale bar, 100 μm; magnification, 20 × and 40 ×). n = 3. ns: *p* > 0.05, * *p* < 0.05, ** *p* < 0.01.

Further *in vivo* experiments were performed to evaluate the influence of PLK4 silencing on tumorigenesis and metastasis. sh-PLK4 or sh-control-transfected cells were subcutaneously implanted into nu/nu mice. Remarkably, the results demonstrated that the HCT8-shPLK4-derived tumors exhibited a slower growth rate than that of control group (Figure 6A). Furthermore, knockdown of PLK4 expression in HCT8 cells significantly decreased tumor volumes and weights in the xenograft mouse model (Figure 6A&B). Moreover, we created lung metastasis mouse models via tail vein injection of sh-PLK4 or sh-control of HCT8 cells. Metastatic tumor nodules were evaluated and quantified by two pathologists examining H&E-stained sections. Interestingly, the incidence of metastatic lesions in the lungs was significantly lower in the sh-PLK4 group than in the sh-control group while there were no significant differences observed in lung volume and weight between the two groups (Figure 6C and Figure S4A-C). These results suggest that decreased PLK4 expression is negatively correlated with the malignant progression of CRC, which may be related to the dormancy of CRC cells.

Considering the regulatory role of PLK4 in CRC dormancy *in vitro*, we further detected the cell cycle and apoptosis in xenograft tumors and compared the results between the sh-PLK4 and sh-control groups. Similar to the *in vitro* results, depletion of PLK4 in CRC cells induced G0/G1 cell cycle arrest without any changes in apoptosis (Figure 6D&E). Notably, increases in the p-p38^high^/p-ERK^low^ signaling ratio, Beclin1, Atg5, LC3B-II expression were observed in tumors established with sh-PLK4 cells, whereas the expression of p62, PCNA, CCND1 and CCNE1 was dramatically decreased without caspase activation (Figure 6F). The depletion of PLK4 significantly increased the mRNA levels of *p15*, *p16* and *p27*, which aligned with the results observed *in vitro* (Figure 6G). In addition, IHC staining for Ki67, p-ERK, p-p38 and LC3B revealed results consistent with the above data (Figure 6H and Figure S3D).

**Figure 7:**
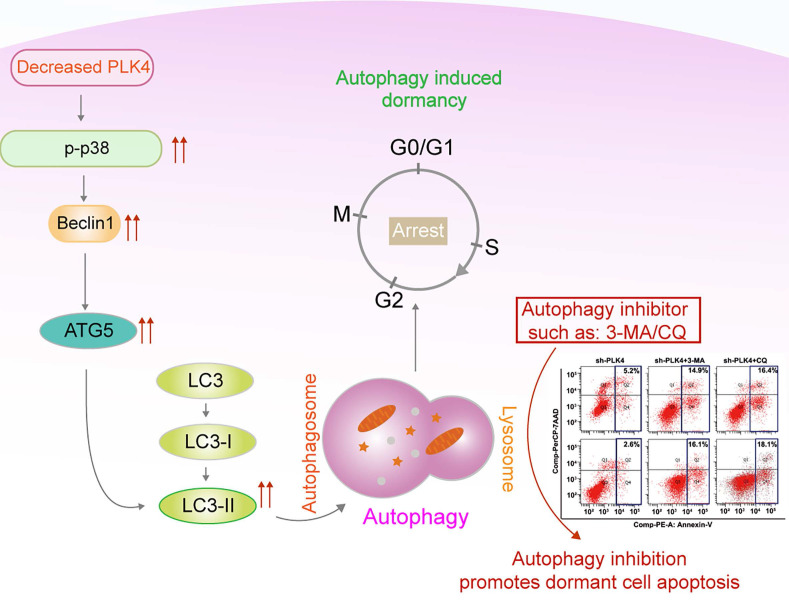
Working model.

Together, these findings demonstrated that knockdown PLK4 in CRC cells inhibited tumor growth and metastasis in xenograft models, further revealing a critical role for PLK4-induced autophagy in the initiation of tumor dormancy. A schematic model illustrating our findings was shown in Figure 7.

## Discussion

Tumor dormancy is a process whereby cells enter reversible G0/G1 arrest and the disease remains in an undetectable state before appearing as an overtly proliferative disease^28^. p38 MAPK^high^/ERK^low^ phenotype is now widely used as a marker of dormant cells and the proliferation marker MKI67 and the cyclin-dependent kinase (CDK) inhibitor P27 are often used to label and identify dormant tumor cells^18^, ^34^. Currently, the pharmacological strategies for targeting dormant tumor cells include three approaches^35^. First is the reactivation of dormant cells reactivated to increase their susceptibility to antiproliferative drugs. For example, forcing quiescent residual leukemic cells to reenter the cell cycle is considered a useful therapeutic strategy for acute myeloid leukemia (AML)^36^. Second is the maintenance of tumor cells in the dormant state. In ER^+^ breast cancers, adjuvant anti-estrogen therapy has been widely used to inhibit dormant cell outgrowth, which effectively prolongs patient survival^37^. As not all dormant cells will respond to reactivation or maintenance, these strategies come with disadvantages that undetectable minimal residual disease will persist^35^-^37^. Third is the eradication of dormant cancer cells and the major concern with this approach is the difficulty to develop novel agents addressing dormant cells^38^, ^39^. This reality emphasizes further the need to develop dormancy-targeting drugs to prevent late metastasis.

The functions of PLK family members, including cell cycle regulation and DNA damage response contributions, as well as participation in the carcinogenesis and metastasis of various cancers, are widely understood^15^, ^40^. Indeed, PLK4, a member of the PLK family, has been clarified to affect the proliferation ability and metastatic potential of cancer cells via distinct mechanism. And abnormal expression of PLK4 contributes to proliferation impairment and cell cycle arrest, which are the two main characteristics of dormant cells according to previous studies^41^, ^42^. Currently, we first functionally delineated that PLK4 knockdown was proved to induce G0/G1 cell cycle arrest in CRC cells, we then mechanistically authenticate that PLK4 serves as a key node between dormancy and proliferation in CRC and downregulation of PLK4 contributes to restoring phenotypically aggressive tumor cells to a dormant state. However, the intrinsic relationship linking PLK4 and dormancy is still unknown.

It is well established that p53 is a key hallmark in CRC, with approximately 40-50% of cases exhibiting mutations in this gene^43^. In addition to its role as a tumor suppressor, p53 is a key regulator of cell cycle and proliferation in CRC^44^. The retinoblastoma protein (Rb) plays a pivotal role in the negative control of the cell cycle and it has been shown that Rb is responsible for a major G1 checkpoint, blocking S-phase entry and cell growth^45^. While HCT-8 CRC cell lines have been reported to contain wildtype p53 (wtp53), SW480 cells harbor mutant p53^46^, ^47^ while mutations in term of Rb on CRC cell lines have yet to be identified. p53 activity is higher in quiescent cells compared with proliferating cells^48^, both the p53-dependent and a p53-independent pathway seem to be an important mechanism in tumor cell dormancy. Dormancy-inducing signals typically produce a decrease in cyclin-CDK activity or an increase in CDK inhibitors or Rb levels^49^. A reduction in RB phosphorylation may stem from a decrease in cyclin D/CDK4 activity^50^. It has been also reported that both cyclin D1 overexpression and related CKI inhibitor alterations produce persistent hyperphosphorylation of pRb, resulting in cell cycle arrest^45^. Tumors with Rb mutation are generally characterized by reduced levels of cyclin D1 or high levels of p16 as they might lie in the same pathway^51^. However, the manner in which G1 regulation is disrupted in dormant colorectal cancer cells is not known. In the current study, we have demonstrated that loss of PLK4 induces a G0/G1 arrest with a decrease in CDK4/CDK6 activity in both HCT8 and SW480 cell lines. Leaving cell cycle regulators aside, this effect is associated with autophagy. In our future research, we would ask whether and how colorectal cancer cells with mutant p53, Rb or APC affected cell cycle progression to determine their role in CRC dormancy.

Though underlying mechanism of cancer dormancy is not fully understood, the relationships between autophagy and tumor dormancy have attracted great attention as a hot topic^52^. Autophagy was involved in cell cycle regulation and proliferation, a study by Washington MN *et al* suggested that in ovarian cancer autophagy stimulation led to a reversible dormant state and inhibition of tumor growth, however, the autophagy inhibitor, chloroquine, reduced tumor growth, emphasizing the importance of autophagy to tumor dormancy^53^. Autophagic machinery primarily relies on the activity of autophagy-related (ATGs) proteins. Generally, MAPK activates autophagy, subsequent formation of phagophore is promoted by activation of Beclin1 or ULK1 complexes^54^. Phagophores eventually forms autophagosomes, which fuse with lysosomes to form autolysosomes. Beclin1 complexes were reported to be the important mediator involved in dormancy-related autophagy^33^. ATG5, ATG7 and p62 were also reported to be critical for autophagy-regulated dormancy^33^, ^55^. Recent studies have clarified that autophagy inhibitor targeting dormant cells would prevent cancer recurrence^33^, ^55^, ^56^. Accordingly, one can speculate that Beclin1 complexes may be vital for maintaining autophagy-regulated dormant state and one fascinating topic for future investigation about autophagy inhibitor is required to directly initiate the death of dormant tumor cells.

In our study, both immunofluorescence and WB assays implied that autophagy was essential for dormancy initiation since Beclin1, Atg5 and LC3B-II strongly increased while p62 level dramatically declined upon PLK4 deficiency. Followed by identification with autophagy inducer, dormancy-associated protein expression levels were inversely regulated. Additionally, dormant CRC cells were likely more sensitive to autophagy inhibition. These results were in line with the data of Vera-Ramirez L *et al*^33^. Thus, PLK4 knockdown induced autophagy is a critical process during initiation and maintenance CRC dormancy. Nevertheless, there are also discrepant findings that autophagy may also lead dormant cells to re-enter into a proliferative phase, fortunately, this dormant-to-proliferative switch renders cancer cells susceptible to traditional antiproliferative strategies. The controversial results may underscore the complicated role of autophagy played in dormancy which requires detailed understanding. Besides, Vera-Ramirez L* et al* observed that autophagy was an early event in breast cancer dormancy and preliminarily illustrated that dormant cells activated a Beclin1-independent autophagy pathway^33^.

Herein, we found p62 level seems to be very sensitive against PLK4 manipulation. P62/SQSTM1 (hereafter called p62) is a versatile scaffolding protein that can integrate and regulate a variety of cellular processes^57^. In autophagy, p62 serves as an autophagy receptor that tags cytoplasmic components for sequestration into an autophagosome^58^. Both transcriptional and post-translational mechanisms, such as phosphorylation and ubiquitination of p62, play critical roles in the regulation of autophagy^58^. Though no direct connection has been established between p62 activity and entry or exit from cellular dormancy, p62/SQSTM1 has been shown to be degraded in several models of dormancy, suggesting that it may be an important mediator between autophagy and dormancy^52^. Some evidence has shown knockdown of p62 results in cell cycle arrest accompanied by entry of cells into dormancy, which suggested that intrinsic modulation of p62 might be a direct mechanism through which tumor cells enter or exit from dormancy in ovarian carcinoma^59^. How PLK4 downregulation affects autophagy and how the autophagy receptors contribute to the PLK4-downregulation induced autophagy in dormant cells need further investigation.

Mitogen-activated kinase (MAPK) pathway is placed at the center of dormancy regulation and activation of p38 pathway supported the involvement of proliferation arrest in several dormant cancer types^18^, ^60^. Besides, MAPK pathway activates autophagy under nutrient deprivation, the activation of p38 MAPK is related to the increased protein level of autophagic markers (such as ATG5 and LC3B)^54^, ^61^. Though a small amount of literature has been reported that MAPK inhibition has effect on PLKs-associated cell viability, few studies had attempted to identify the relationship between PLK4 expression and MAPK signaling pathway^62^. In the present study, we found that p-p38 expression changed following PLK4 expression. BIRB796, the inhibitor of the MAPK signaling pathway could dramatically inhibit autophagy and dormancy while increase apoptosis in PLK4-knockdown cells. Collectively, PLK4 may induce autophagy and regulate dormancy through the MAPK signaling pathway in CRC cells.

In total, we confirmed that PLK4 downregulation regulated cell dormancy by promoting autophagy through the MAPK signaling pathway and the apoptosis rate evidently increased in dormant CRC cells treated with autophagy inhibitor 3-MA. This study enriches the functional value of PLK4 in hallmarks of tumors, and opens up potential avenues for autophagy inhibitor as potential dormancy therapeutic agents for CRC therapy. Therefore, it will be of interest in the future as more potential therapies are developed to eradicate dormant tumor cells by autophagy inhibition, ultimately reducing late relapse in CRC patients.

## Supplementary Material

Supplementary figures.

## Figures and Tables

**Table 1:** The primer sequences in this manuscript

	F-primer	R-primer
***PLK4***	GTACGATTCTGATAACCCC	GGGGTTATCAGAATCGTAC
***MKI67***	CTGACCCTGATGAGAGTGAGGG	TCTCCCCTTTTGAGAGGCGT
***p15***	TGGGGTGGGAAAGTGGATTGCA	CCCAGTGCAGAGGTGTTCAGGTCT
***p16***	GCTGCTCACCTCTGGTGCCAAA	ACCTGCGCACCATGTTCTCG
***p27***	GGTTAGCGGAGCAATGCGCA	AACCGGCATTTGGGGAACCGTC
***LRG1***	GGGTCAGAACCAAGGGGTTT	GTCACAGGCCATTGATCCCA
***BMP7***	ACGAGGTGCACTCGAGCTT	GAAGCGTTCCCGGATGTAGT
***Beclin1***	CTGGACACTCAGCTCAACGTCA	CTCTAGTGCCAGCTCCTTTAGC
***Atg5***	GCAGATGGACAGTTGCACACAC	GAGGTGTTTCCAACATTGGCTCA
***LC3B***	GAGAAGCAGCTTCCTGTTCTGG	GTGTCCGTTCACCAACAGGAAG
***p62***	TGTGTAGCGTCTGCGAGGGAAA	AGTGTCCGTGTTTCACCTTCCG
***GAPDH***	TGGTATCGTGGAAGGACTCA	CCAGTAGAGGCAGGGATGAT

**Table 2:** Univariate and multivariate analysis of clinical parameters on overall survival of CRC patients

Clinicopathologic features	Univariate analysis				Multivariate analysis		
	HR (log rank)	95% CI	*p* value		HR (log rank)	95% CI	*p* value
**Sex (male/female)**	0.986	0.511-1.903	0.967				
**Age (>55/≤55 years old)**	1.316	0.648-2.676	0.446				
**CEA (>5/≤5 μg/L)**	3.211	1.586-6.500	0.001		2.392	1.142-5.010	0.021
**Tumor size (>5/≤5 cm)**	0.747	0.388-1.435	0.381				
**Vascular invasion (yes/no)**	0.589	0.258-1.346	0.210				
**Lymph nodes metastasis (yes/no)**	0.402	0.209-0.774	0.006		0.696	0.220-2.204	0.537
**Tumor capsule (yes/no)**	0.364	0.159-0.831	0.016		0.692	0.270-1.775	0.444
**Tumor location (left/right)**	0.519	0.267-1.006	0.048		0.605	0.297-1.234	0.167
**TNM stage (III-IV/I-II)**	2.251	1.140-4.447	0.019		0.812	0.252-2.614	0.727
**PLK4 expression (high/low)**	3.186	1.627-6.240	<0.001		2.465	1.157-5.250	0.019
**Ki67 expression (high/low)**	2.188	1.108-4.322	0.024		1.356	0.614-2.996	0.451

**Table 3:** The correlations between PLK4 and various clinicopathological factors in CRC patients

Characteristics		Total	PLK4 expression		*p* value	Characteristics		Total	PLK4 expression		*p* value
		122	Low	High				122	Low	High	
**Sex**					0.580	**LN metastasis**					0.012*
	Male	56	38	28			yes	43	19	24	
	Female	66	35	21			no	79	54	25	
**Age**					0.380	**Tumor capsule**					0.001*
	>55	79	45	34			no	78	38	40	
	≤55	43	28	15			yes	44	35	9	
**CEA**					0.034*	**Tumor location**					0.853
	>5	46	22	24			left	55	32	23	
	≤5	76	51	25			right	67	41	26	
**Tumor size**					0.041*	**TNM stage**					0.002*
	>5	55	27	28			III-IV	59	27	32	
	≤5	67	46	21			I-II	63	46	17	
**VI**					0.180	**Ki67 expression**					<0.001*
	yes	16	7	9			high	59	25	34	
	no	106	66	40			low	63	48	15	

## Abbreviations

AJCC: American Joint Committee on Cancer
CRC: colorectal cancer
CDK: cyclin-dependent kinase
IHC: Immunohistochemistry
PLK4: Polo-like kinases 4
WB: western blotting
qRT-PCR: Quantitative real time polymerase chain reaction
