# Supplementary material for: Autophagy Inhibition Contributes to Apoptosis of PLK4 Downregulation-induced Dormant Cells in Colorectal Cancer
Source: Int J Biol Sci. 2023 May 29;19(9):2817–34. doi: 10.7150/ijbs.79949 (PMC10266079; doi:10.7150/ijbs.79949)
Supplement: Supplementary file 1 — Supplementary figures. [file ijbsv19p2817s1.pdf]

Figure S1. PLK4 affects proliferation and invasion of CRC cells.

A. The verification of PLK4-control (sh-control), PLK4-knockdown (sh-PLK4) and empty-vector and PLK4-overexpresison (PLK4-OE) in CRC cell lines. B. CCK-8 assay revealing the proliferative abilities of SW480 and HCT8 cells in the sh-control and sh-PLK4 groups. C. GSEA showing the relationships between PLK4 expression and proliferation as well as cell cycle genes. D&E. Wound-healing, chemotaxis assays comparing cell migration and invasion differences between the sh-control and sh-PLK4 groups of SW480 and HCT8 cells (scale bar, 1.0 mm). F. The SA-β-gal staining for senescence cells in sh-control and sh-PLK4 groups and the percentage of SA-β-gal positive cells were quantified (scale bar, 150 μm). ns: $p > 0.05$, * $p < 0.05$, ** $p < 0.01$, *** $p < 0.001$.

Figure S2. PLK4 is overexpressed in CRC tissues and associated with a series of malignant behaviors linked to an unfavorable prognosis.

A-F: GSEA was used to analysis the associations of high PLK4 expression in CRC with a series of malignant behaviors linked to an unfavorable prognosis in CRC.G. Enhanced PLK4 expression was observed in tumor tissues compared with adjacent non-tumor tissues in most cancer types, including CRC. H. The expression of PLK4 was examined in CRC tissue samples and adjacent nonmalignant tissue samples from the TCGA and GEO databases (N: normal, T: tumor, and P: polyp). I-L: Correlation analysis between the PLK4 IHC score and lymphatic metastasis, tumor capsule, TNM stage as well as CEA in the CRC cohort. M-P. Kaplan-Meier survival curve showing the correlation between the PLK4 IHC score and OS of the high-risk subgroup in the CRC cohort. **

$p < 0.01$, *** $p < 0.001$.

Figure S3. A. The representative flow cytometry images of cell cycle in Vector, PLK4 OE with or without rapamycin groups. B. The mRNA levels of PLK4 and autophagy-related markers in sh-control, sh-PLK4 with or without BIRB796. ns: $p > 0.05$, * $p < 0.05$, ** $p < 0.01$, *** $p < 0.001$.

Figure S4. A-C. The lung images (A), lung weights (B), and H&E staining of lung metastasis in tail vein-injected mice (scale bar, 100 μm; magnification, 4 × and 40 ×) D. Verification of PLK4 and cell proliferation, cell cycle and autophagy markers in the xenograft model, as assessed by IHC (scale bar, 100 μm; magnification, 20 × and 40 ×). ns: $p > 0.05$.

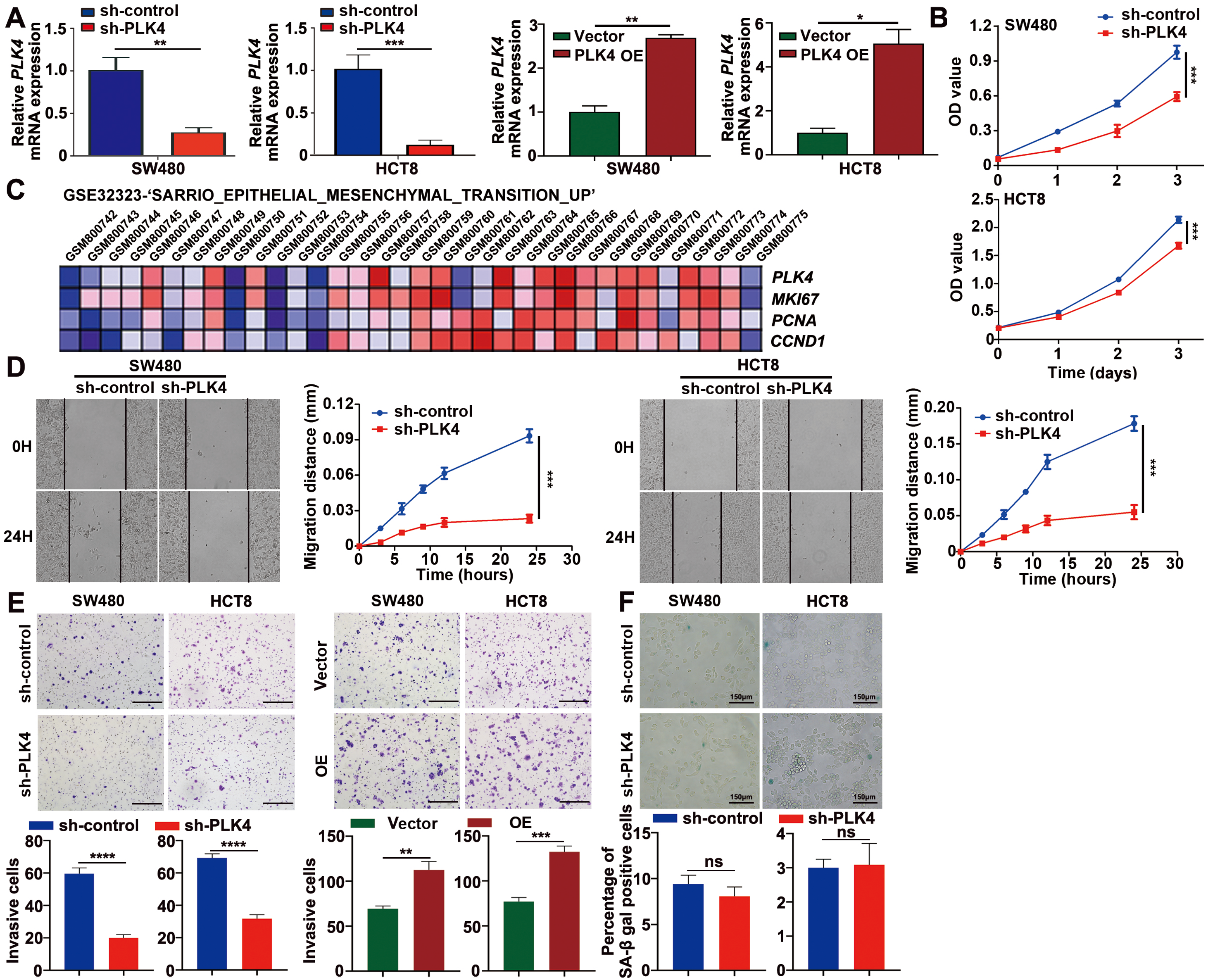
A
sh-control
sh-PLK4
Relative PLK4 mRNA expression
SW480
HCT8
Vector
PLK4 OE
B
SW480
HCT8
OD value
Time (days)
C
GSE32323-'SARRIO_EPITHELIAL_MESENCHYMAL_TRANSITION_UP'
PLK4
MKI67
PCNA
CCND1
D
0H
24H
Migration distance (mm)
Time (hours)
E
Vector
OE
Invasive cells
F
150μm
Percentage of SA-β gal positive cells
ns

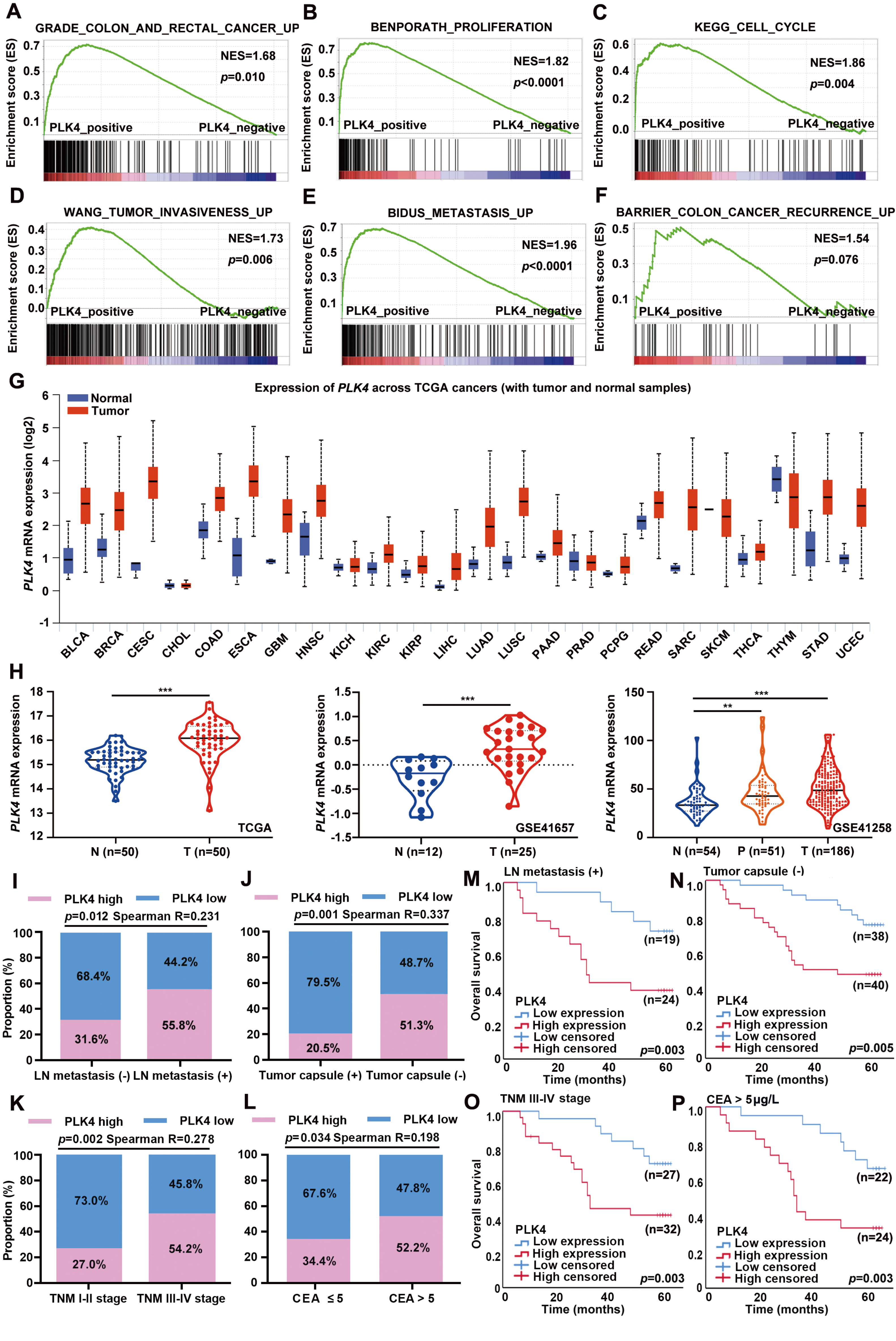
A
GRADE_COLON_AND_RECTAL_CANCER_UP
NES=1.68
p=0.010
PLK4_positive
PLK4_negative
Enrichment score (ES)
B
BENPORATH_PROLIFERATION
NES=1.82
p<0.0001
PLK4_positive
PLK4_negative
C
KEGG_CELL_CYCLE
NES=1.86
p=0.004
PLK4_positive
PLK4_negative
D
WANG_TUMOR_INVASIVENESS_UP
NES=1.73
p=0.006
PLK4_positive
PLK4_negative
E
BIDUS_METASTASIS_UP
NES=1.96
p<0.0001
PLK4_positive
PLK4_negative
F
BARRIER_COLON_CANCER_RECURRENCE_UP
NES=1.54
p=0.076
PLK4_positive
PLK4_negative
G
Expression of PLK4 across TCGA cancers (with tumor and normal samples)
Normal
Tumor
PLK4 mRNA expression (log2)
BLCA BRCA CESC CHOL COAD ESCA GBM HNSC KICH KIRC KIRP LIHC LUAD LUSC PAAD PRAD PCPG READ SARC SKCM THCA THYM STAD UCEC
H
PLK4 mRNA expression
TCGA
N (n=50)
T (n=50)
GSE41657
N (n=12)
T (n=25)
GSE41258
N (n=54)
P (n=51)
T (n=186)
I
PLK4 high
PLK4 low
p=0.012 Spearman R=0.231
Proportion (%)
68.4%
31.6%
44.2%
55.8%
LN metastasis (-) LN metastasis (+)
J
PLK4 high
PLK4 low
p=0.001 Spearman R=0.337
79.5%
20.5%
48.7%
51.3%
Tumor capsule (+) Tumor capsule (-)
M
LN metastasis (+)
Overall survival
(n=19)
(n=24)
PLK4
Low expression
High expression
Low censored
High censored
p=0.003
Time (months)
N
Tumor capsule (-)
(n=38)
(n=40)
PLK4
Low expression
High expression
Low censored
High censored
p=0.005
Time (months)
K
PLK4 high
PLK4 low
p=0.002 Spearman R=0.278
Proportion (%)
73.0%
27.0%
45.8%
54.2%
TNM I-II stage TNM III-IV stage
L
PLK4 high
PLK4 low
p=0.034 Spearman R=0.198
67.6%
34.4%
47.8%
52.2%
CEA ≤5
CEA > 5
O
TNM III-IV stage
Overall survival
(n=27)
(n=32)
PLK4
Low expression
High expression
Low censored
High censored
p=0.003
Time (months)
P
CEA > 5μg/L
(n=22)
(n=24)
PLK4
Low expression
High expression
Low censored
High censored
p=0.003
Time (months)

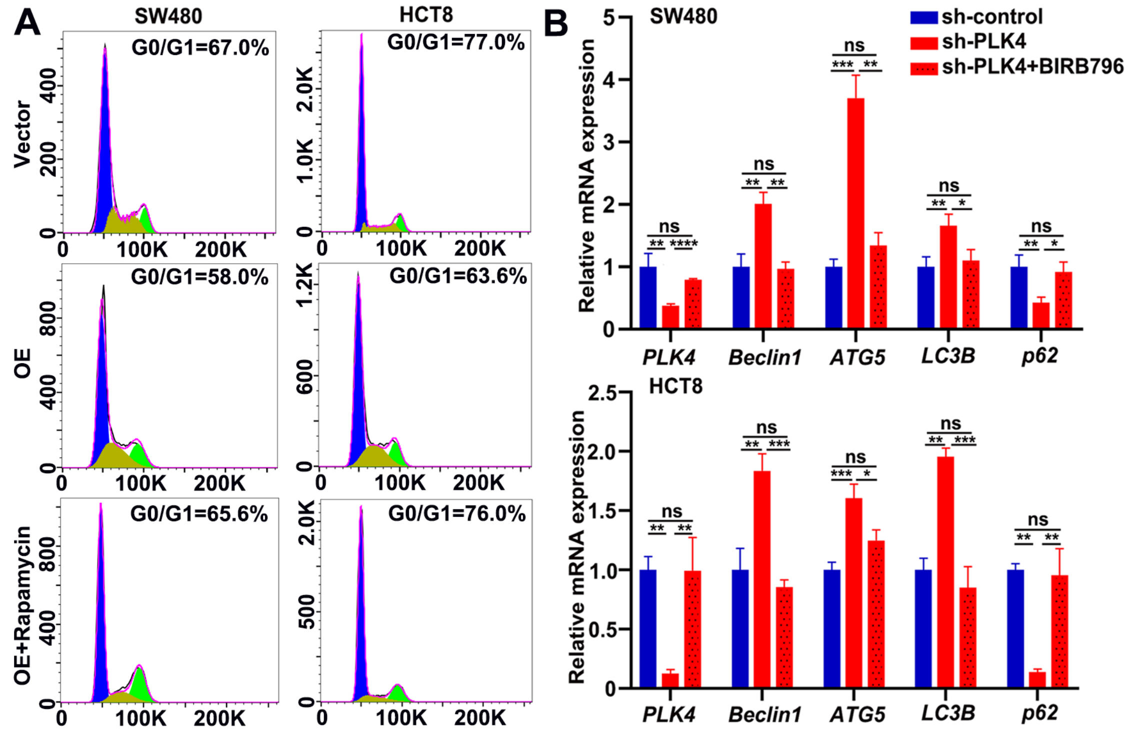
A
SW480
HCT8
Vector
OE
OE+Rapamycin
G0/G1=67.0%
G0/G1=77.0%
G0/G1=58.0%
G0/G1=63.6%
G0/G1=65.6%
G0/G1=76.0%
0
100K
200K
B
SW480
HCT8
sh-control
sh-PLK4
sh-PLK4+BIRB796
Relative mRNA expression
PLK4
Beclin1
ATG5
LC3B
p62
ns

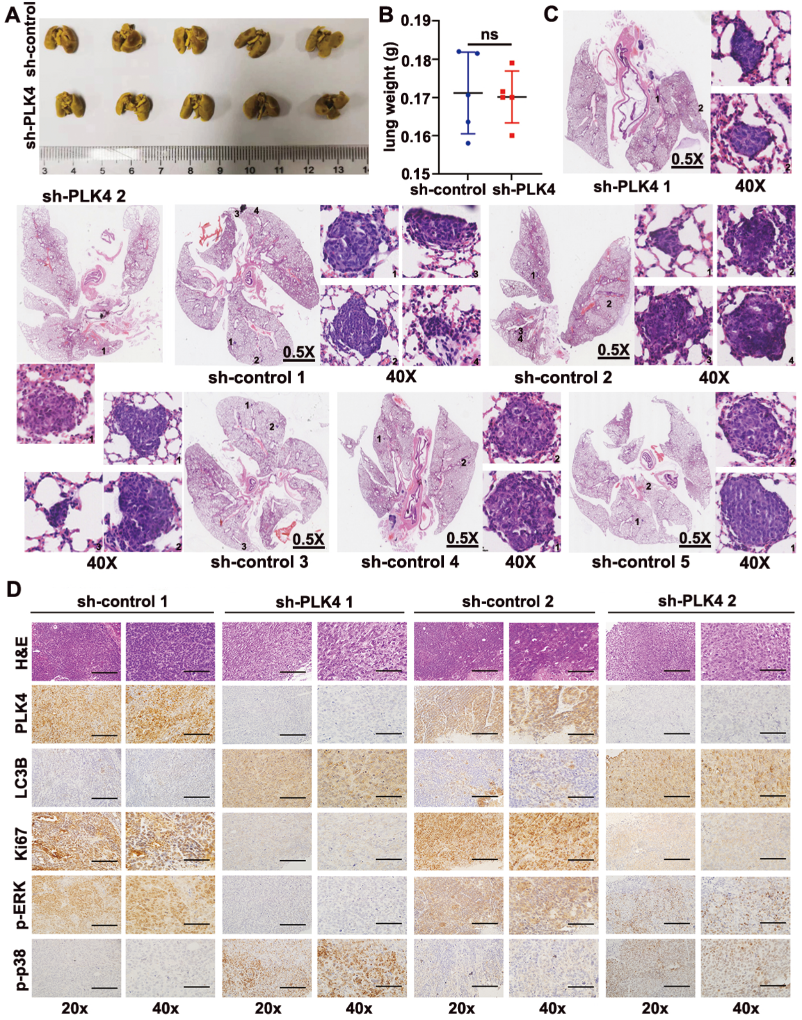

A
sh-control
sh-PLK4
B
lung weight (g)
0.19
0.18
0.17
0.16
0.15
ns
sh-control
sh-PLK4
C
0.5X
sh-PLK4 1
40X
sh-PLK4 2
sh-control 1
40X
sh-control 2
40X
40X
sh-control 3
sh-control 4
40X
sh-control 5
40X
D
sh-control 1
sh-PLK4 1
sh-control 2
sh-PLK4 2
H&E
PLK4
LC3B
Ki67
p-ERK
p-p38
20x
40x
20x
40x
20x
40x
20x
40x
